# Functionalized Magnetic Fe_3_O_4_ Nanoparticles for Targeted Methotrexate Delivery in Ovarian Cancer Therapy

**DOI:** 10.3390/ijms25169098

**Published:** 2024-08-22

**Authors:** Julia Nowak-Jary, Artur Płóciennik, Beata Machnicka

**Affiliations:** 1 Department of Biotechnology, Institute of Biological Sciences, University of Zielona Gora, 65-516 Zielona Gora, Poland; b.machnicka@wnb.uz.zgora.pl; 2 Institute of Experimental Biology, University of Poznan, 61-614 Poznan, Poland; artur.plociennik@amu.edu.pl

**Keywords:** magnetic iron oxide nanoparticles, methotrexate, ovarian cancer, cytotoxicity

## Abstract

Magnetic Fe_3_O_4_ nanoparticles (MNPs) functionalized with (3-aminopropylo)trietoksysilan (APTES) or N-carboxymethylchitosan (CMC) were proposed as nanocarriers of methotrexate (MTX) to target ovarian cancer cell lines. The successful functionalization of the obtained nanostructures was confirmed by FT-IR spectroscopy. The nanoparticles were characterized by transmission electron spectroscopy (TEM) and dynamic light scattering (DLS) techniques. Their potential zeta, magnetization, and hyperthermic properties were also explored. MTX was conjugated with the nanocarriers by ionic bonds or by amide bonds. The drug release kinetics were examined at different pH and temperatures. The MTT assay showed no toxicity of the MNPs[APTES] and MNPs[CMC]. Finally, the cytotoxicity of the nanostructures with MTX attached towards the ovarian cancer cells was measured. The sensitivity and resistance to methotrexate was determined in simplistic 2D and spheroid 3D conditions. The cytotoxicity tests of the tested nanostructures showed similar values for inhibiting the proliferation of ovarian cancer cells as methotrexate in its free form. Conjugating MTX with nanoparticles allows the drug to be directed to the target site using an external magnetic field, reducing overall toxicity. Combining this approach with hyperthermia could enhance the therapeutic effect in vivo compared to free MTX, though further research on advanced 3D models is needed.

## 1. Introduction

Due to advances in nanotechnology, magnetic iron oxide nanoparticles (MNPs) (magnetite Fe_3_O_4_) are now being extensively used for making medicines. The uniqueness of MNPs comes from their ferromagnetic nature as they consist of areas of permanent magnetization (so-called magnetic domains), generating a magnetic field around them (like small magnets). Because of their magnetism, simple synthesis and functionalization, large specific surface area, biocompatibility, biodistribution, and physicochemical properties, MNPs are employed in, i.e., tumour imaging (MRI) [1,2,3], radiolabelling, internal radiotherapy [4], hyperthermia [5,6], gene therapy [7], drug delivery and theranostics [8,9]. Magnetic nanoparticles have been considered feasible candidates for developing novel antitumour therapeutics with the potential to overcome the drug resistance of cancer cells.

Resistance to cytotoxic drugs accounts for 90% of cancer deaths [10]. Cancer cell heterogeneity and mutations lead to multidrug resistance (MDR), enhancing survival in the presence of drugs. These mechanisms include reduced drug influx, increased excretion, drug metabolism, DNA repair, lack of apoptosis, and increased anti-apoptotic activity. MDR mechanisms can be cellular or tissue-specific [11]. ABC transporter family proteins, like P-glycoprotein (MDR1), breast cancer resistance protein (BCRP), and multidrug resistance protein 1 (MRP1) regulate chemical distribution, absorption, and excretion. Overexpression of these transporters lowers the intracellular cytostatic concentration [10,12]. For example, excess BCRP and MRP1 proteins are linked to methotrexate resistance [13]. Moreover, cancers interact with their microenvironment, including blood and lymphatic vessels, stromal cells, and the extracellular matrix, leading to cytostatic resistance [14]. The tumour’s atypical vasculature and hypoxic areas hinder drug delivery and efficacy by regulating drug-resistance genes and triggering specific signalling pathways such as NFĸB, MAPK, PI3K, and HIF [15,16]. Atypical vessels create acidic environments affecting cytostatic release, and lymphatic abnormalities weaken immune responses. Immune cells like cancer-associated fibroblasts (CAF) and macrophages contribute to resistance by interacting with drugs and producing cytokines that block T-cell activation [17]. The dense tumour matrix prevents uniform drug delivery, with resistance linked to its structure [15]. Cell adhesion-mediated drug resistance (CAM-DR) involves interaction with the tumour matrix via β1 integrin, as seen in various cancers [18]. Besides, cancer stem cells (CSCs) add to resistance through self-renewal, drug resistance protein expression, and signalling pathway regulation [11,19].

Some of the phenomena mentioned above specific to tumours in vivo can be observed in three-dimensional cell models [20]. Adherently growing cells lose their polarity, which changes the response of these cells to various phenomena, e.g., apoptosis [21]. It has also been observed that culture in 2D conditions changes gene expression and splicing, cell topology, and biochemistry [22,23,24]. Culturing cancer cells in 3D involves creating spheroidal structures that mimic the physical and biochemical aspects of the tumor mass. In this cellular model, intercellular and cell-environment interactions are similar to those in vivo [25,26]. The natural morphology, polarity, cell topology, gene expression, signalling, and metabolism are more similar to in vivo conditions [27].

To overcome the drug resistance to methotrexate in ovarian cancer cells, we designed nanoparticles with the drug attached. First, Fe_3_O_4_ nanoparticles functionalized with APTES or CMC were synthesized. The proper coating of the Fe_3_O_4_ core increases the biocompatibility of magnetic nanocarriers and reduces their potential toxicity. The polymer shell minimizes free iron ion release. Free iron ions are believed to participate in the Haber–Weiss, Fenton, and Fenton-like reactions, the main processes generating reactive oxygen species (ROS) such as superoxide anions, hydroxyl radicals, and hydrogen peroxides [28]. Additionally, coating molecules can provide various functional groups as linkers for therapeutic attaching. As an example, aminosilanes, including 3-(aminopropyl)triethoxysialne (APTES) [29,30], are frequently used for MNPs functionalization since they provide amine groups for drug binding. Other often applied biocompatible polymers are chitosan and its derivatives, such as N-carboxymethylchitosan (CMC) equipped with carboxyl groups [31]. These molecules are also responsible for the resultant MNPs charge, influencing their stability in the media. It is worth mentioning that CMC has much better solubility in aqueous media than chitosan and efficiently interacts with cells/tissues; thus, its molecules are used in drug delivery as anti-infective, anticancer, antitumor, antioxidant, and anti-inflammatory agents [32]. An additional advantage of using APTES and CMC is that the polymerization reaction of these molecules around MNPs takes place in quite mild conditions and is simple, efficient, and does not generate high costs.

This paper is dedicated to a detailed description and evaluation of the potential of the functionalized magnetic Fe_3_O_4_ nanoparticles we have developed for the targeted delivery of methotrexate in ovarian cancer cells. Here, we designed, synthesized, and detailed characterized nanoparticles coated by (3-aminopropylo)trietoksysilan (APTES) or N-carboxymethylchitosan (CMC) and with methotrexate (MTX) attached by two kinds of bonds—ionic or amide. We used 2D monolayer and 3D spheroid models, accepted in cancer research for their ability to mimic the in vivo tumor microenvironment, to study the cytotoxicity of these magnetic nanostructures conjugated with MTX against ovarian cancer cells. During this investigation, qualitative and quantitative methods were used to assess first the characteristics of the obtained nanostructures and then their cytotoxicity. To our best knowledge, no studies have been carried out using methotrexate carriers designed by us against sensitive and resistant ovarian cancer cells in 2D and 3D model conditions.

Our study aimed to investigate the use of functionalized magnetic Fe_3_O_4_ nanoparticles as a targeted delivery system for methotrexate (MTX) in treating ovarian cancer. The ultimate research purpose is to enhance the effectiveness of MTX treatment by leveraging the unique magnetic properties of MNPs.

## 2. Results

### 2.1. Characterization with FT–IR and Transmission Electron Microscope (TEM) Imaging of APTES- and CMC-Modified Nanoparticles (MNPs[APTES] and MNPs[CMC])

**Figure 1:**
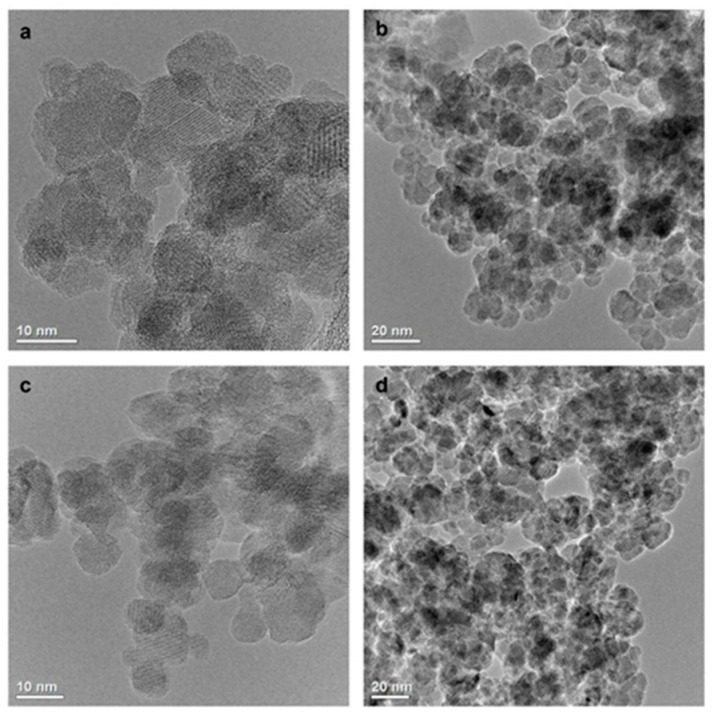
TEM images of MNPs[APTES] (**a**,**b**) and MNPs[CMC] (**c**,**d**).

**Figure 2:**
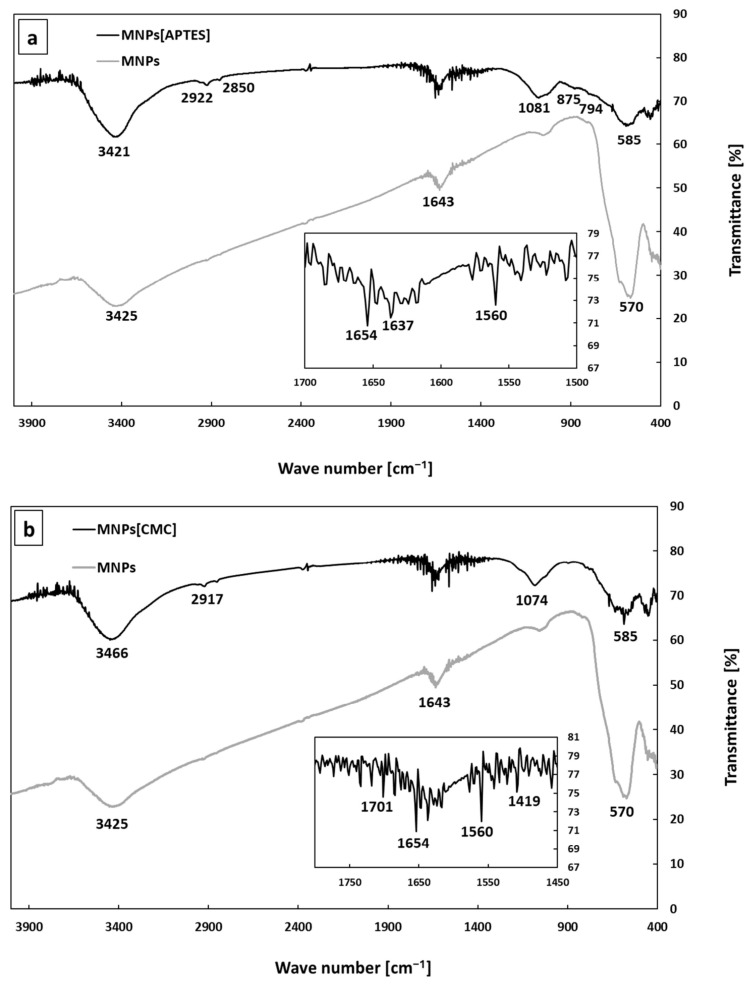
FT–IR spectra of MNPs. MNPs[APTES] (**a**) and MNPs[CMC] (**b**).

The TEM images (Figure 1) show that the MNPs[APTES] and MNPs[CMC] have a uniform shape and size distribution with a diameter of approximately 10 nm. The effect of the surface functionalization of the bare MNPs and MNPs coated with APTES and CMC, respectively, was confirmed by the FT–IR spectrum in the range of 4000–400 cm^−1^ (Figure 2a,b). The intensive spectrum band at 570 cm^−1^ for bare MNPs represents stretching vibrations of Fe–O in the crystalline lattice of Fe_3_O_4_. In contrast, the bands at 3425 cm^−1^ and 1643 cm^−1^ refer to non-dissociated -OH groups present on the MNPs surface after stabilization with TMAOH. The absorption band at 585 cm^−1^ for the MNPs[APTES] sample is associated with the metal-oxygen Fe–O bonds (stretching vibrations). The broadband centred at 3421 cm^−1^ derives from N–H stretching vibrations of APTES. The absorption bands at 2922 cm^−1^ and 2850 cm^−1^ correspond to carbon aliphatic chains of C–H (stretching vibrations), whereas these between 1650–1560 derive from N–H deformation vibrations. The band at 1081 cm^−1^ comes from C–N stretching vibrations and overlaps with Si–O asymmetry stretching vibrations, whereas the bands at 875 and 794 cm^−1^ correspond to Si–O symmetry stretching vibrations. The absorption band at 585 cm^−1^ in the MNPs[CMC] sample corresponds to the Fe–O bonds in the crystalline lattice of Fe_3_O_4_ (stretching vibrations). The broad band at 3466 cm^−1^ derives from the stretching vibrations of N–H and O–H bonds, whereas the bands at 2917 and 2850 cm^−1^ are attributed to the C–H stretching vibration bonds. The spectrum bands at 1654 and 1560 cm^−1^ present for both MNPs[APTES] and MNPs[CMC] represent deformation vibrations of N–H, and in turn those at 1074 cm^−1^ are stretching vibrations of C–N. The band at 1419 cm^−1^ displays symmetrical stretching vibrations of O–H (COOH), whereas the band at 1701 cm^−1^ is stretching vibrations of C=O (COOH). As observed, the spectra of MNPs[APTES] and MNPs[CMC] are very similar due to having many of the same types of bonds.

### 2.2. Magnetic VSM Properties Measurements of MNPs[APTES] and MNPs[CMC]

**Figure 3:**
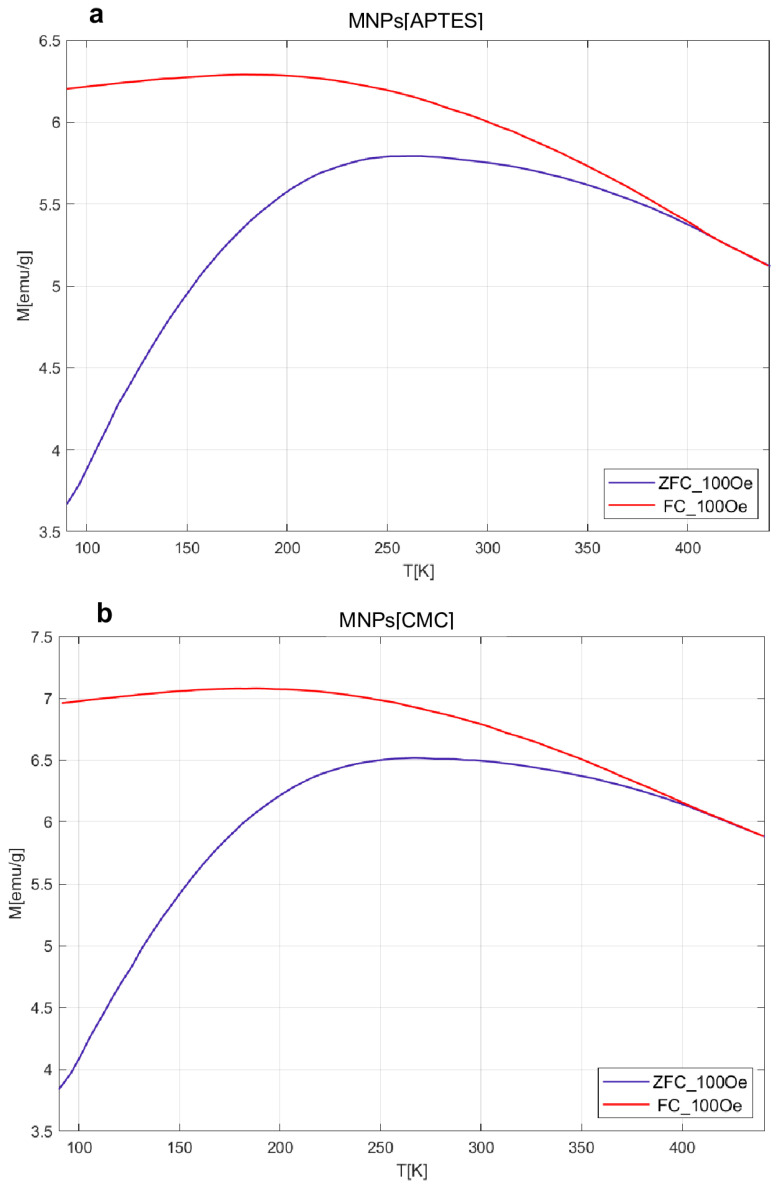
M(T) relationships of the ZFC and FC type for the APTES sample (**a**) and CMC (**b**).

**Figure 4:**
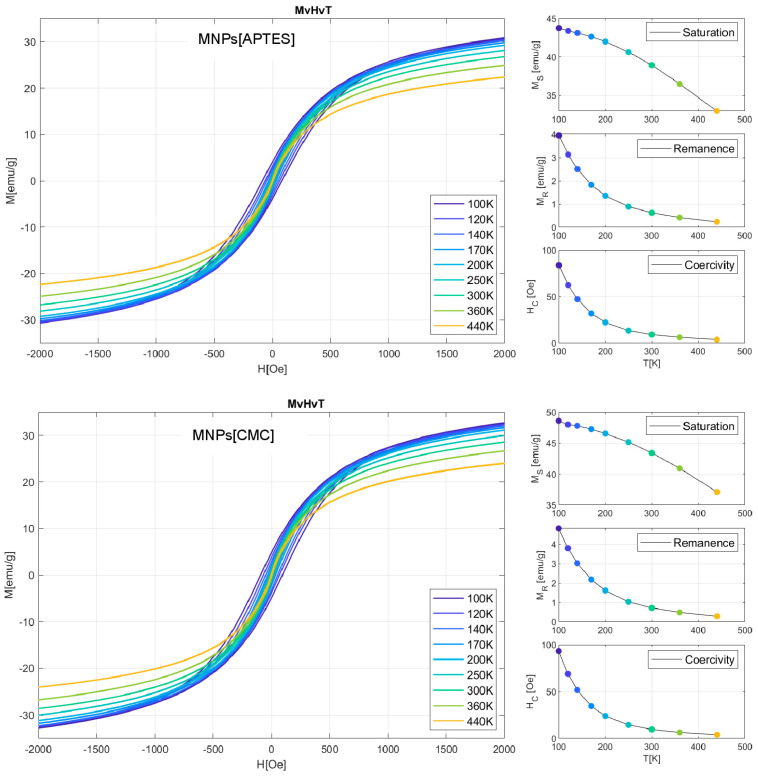
M(H) dependencies for nine selected temperatures in the range from 100 K to 440 K determined on the basis of magnetization saturation, remanence, and coercivity field. The results for the MNPs[APTES] sample are presented in the upper panel and for the MNPs[CMC] sample in the lower panel.

The results obtained for the MvT measurements are shown in Figure 3. For both samples, they indicate a superparamagnetic nature with a blocking temperature (T_B_) estimated from the point inflections of the dM/dT curve at 103 K for the MNPs[CMC] and 113 K for the MNPs[APTES] nanoparticles. Comparisons of the shapes of the measured loops and field relationships coercivity (H_C_), magnetization saturation (M_S_), and magnetic remanence (M_R_) as a function of temperature are presented in Figure 4. The M_S_(T) relationship is typical for a ferrimagnetic iron oxide spinel structure. The nature of the H_C_(T) and M_R_(T) relationships are akin to each other, which suggests that the dominant source of magnetic hysteresis is the blocking effect of single-domain particles when the energy volume anisotropy is higher than the energy of the thermal excitations.

### 2.3. Hyperthermic Properties of MNPs[APTES] and MNPs[CMC]

**Figure 5:**
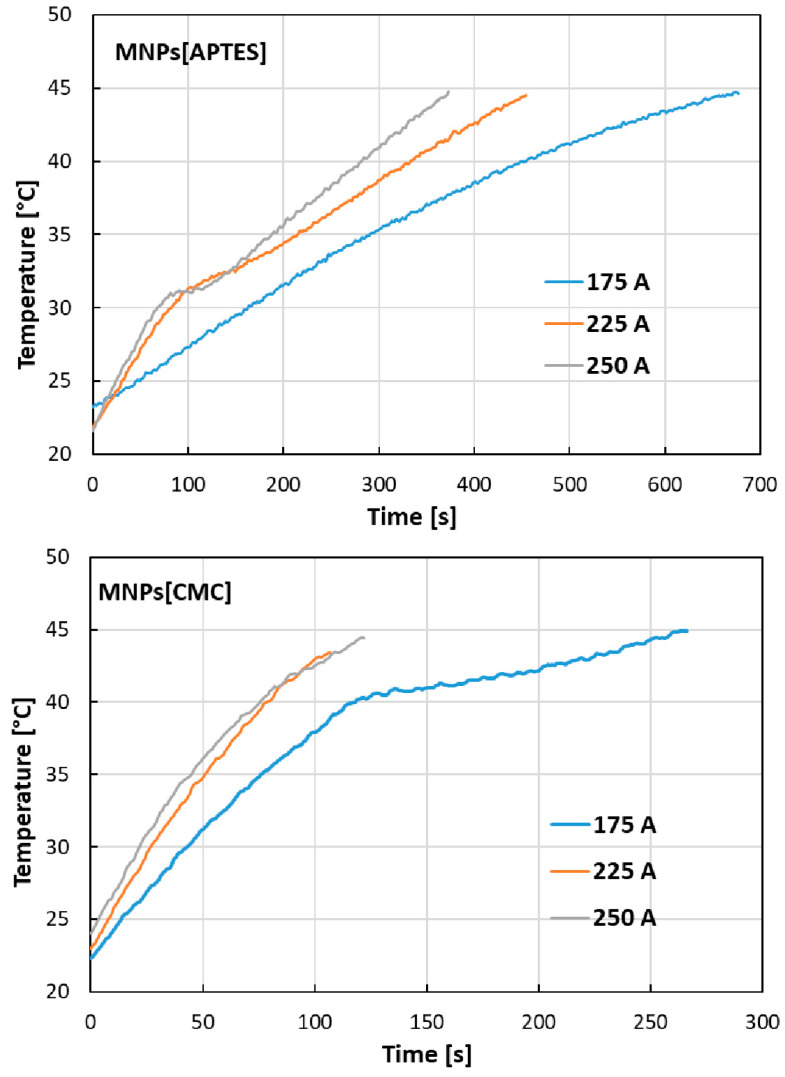
Dependencies of temperature on heating time for MNPs[APTES] and MNPs[CMC] samples for different current values (frequency of magnetic field approx. 360 MHz).

At the given current intensities, the temperature generated by nanoparticles under the influence of an external electromagnetic field increases over time, as shown in Figure 5. The results indicate significant hyperthermal properties of the tested nanostructures, whereby MNPs[CMC] are stronger than MNPs[APTES]. For example, at a current of 250 A, MNPs[APTES] generate a temperature increase to 45 °C after about 370 s, while MNPs[CMC] raise the temperature to the same value after about 120 s.

Based on the measurements obtained using magnetic hyperthermia equipment, the SAR (specific absorption rate) and ILP (intrinsic loss) parameters [33,34,35] were determined and summarized in Table 1. SAR quantifies the ability of magnetic nanoparticles to absorb energy from an alternating magnetic field and can be described by the following equation:

$$SAR=C\times \frac{dT}{tt}\times \frac{1}{m_{Fe}}\;[W/g]$$

where C is the heat capacity of the medium (for water C = 4.189 J/g·K), T is the temperature [K], t is time [s], and m_Fe_ is the mass of iron in the sample. The dT/dt element is determined by fitting a straight line to the initial rectilinear fragment of the temperature vs. time on the graph and reading the slope coefficient of this line. The ILP parameter additionally considers the intensity and frequency of the magnetic field. Its value is calculated using the following equation:

$$ILP=\frac{SAR}{f\times H^{2}}\;\left[{nHm}^{2}/\mathrm{kg}\right]$$

where H is the magnetic field intensity and f is the magnetic field frequency.

SAR and ILP values for induction heating of MNPs[APTES] nanoparticles in an alternating magnetic field increased with the increase in the electromagnetic field intensity (175, 225, and 250 A). In the case of inductive heating of MNPs[CMC] nanoparticles in an alternating magnetic field, the SAR parameter value also increased with the increase in the electromagnetic field strength; however, the SAR and ILP values determined for MNPs[CMC] were several times higher compared to the values determined for the MNPs[APTES] nanoparticles.

### 2.4. Capability of MTX Loading

**Figure 6:**
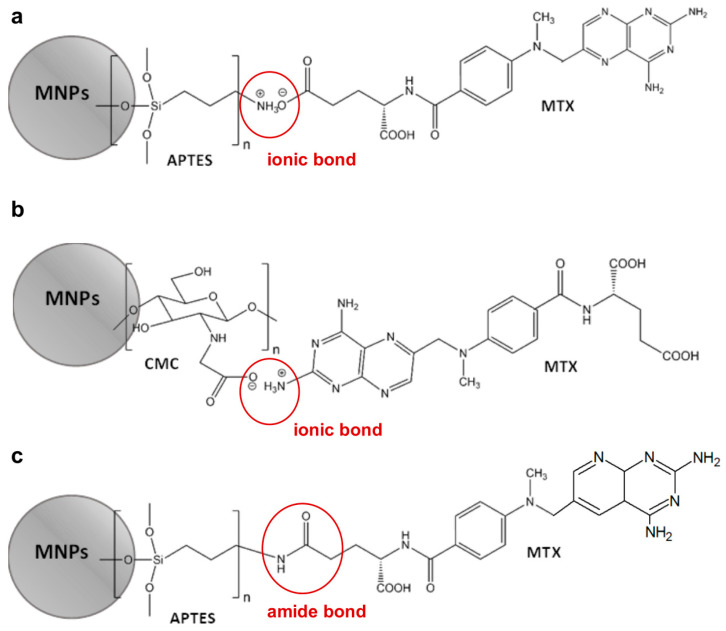
Methods for attaching MTX. MNPs[APTES]—ionic bond (**a**), MNPs[CMC]—ionic bond (**b**), MNPs[APTES]—amide bond (**c**).

Illustrative schemes of methods for attaching MTX to magnetic nanoparticles functionalized with APTES and CMC are illustrated in Figure 6.

Considering that 30 mg of MTX were added to 600 mg of MNPs[APTES] (ionic bond) or MNPs[CMC] (Experimental part), 100% efficiency of loading was theoretically 50 µg MTX per 1 mg of MNPs[APTES]MTX or MNPs[CMC]MTX, respectively. The results of MTX loading capacity quantitation showed that 1 mg of MNPs[APTES]MTX contained 31 µg of MTX, which gave a capability of drug loading equal to 62%, whereas 1 mg of MNPs[CMC]MTX contained 43.6 µg of MTX, which gave a capability of drug loading equal to 87.2%. In the case of MTX conjugated with MNPs[APTES] via an amide bond, the loading efficiency was 18.8% (9.4 µg MTX per 1 mg of nanoparticles).

### 2.5. DLS and Zeta Potential Measurements of MNPs[APTES], MNPs[CMC] and MTX-Functionalized Nanoparticles (MNPs[APTES]MTX and MNPs[CMC]MTX)

The functionalization of MNPs with APTES and CMC and the presence of MTX on the nanoparticles’ surface was further confirmed by the change in surface charge. The ζ-potential of the bare MNPs was −18.8 mV (SD = 0.44). Following coating with APTES and CMC, the charge of the nanoparticles’ surface changed to values equal to +8.4 mV (SD = 0.39) and −18.9 mV (SD = 1.33), respectively. After conjugation of MTX, the nanoparticles exhibited the following ζ-potential values: MNPs[APTES]MTX (ionic bond) −7.4 mV (SD = 0.06), MNPs[CMC]MTX −20.3 mV (SD = 0.74) and MNPs[APTES]MTX (amide bond) −6.1 mV (SD = 0.36). The hydrodynamic diameters (d_H_) of the nanoparticles were determined by the DLS method. The hydrodynamic diameter includes the nanoparticle together with all of its solvated ions from the environment in which they are suspended; therefore, its value is usually much more significant than that determined by electron microscopy, such as with TEM [36]. The obtained D_H_ values in the buffer with a composition corresponding to RPMI 1640 medium were as follows: bare MNPs ~90 nm, MNPs[APTES] ~260 nm, MNPs[CMC] ~36 nm, MNPs[APTES]MTX (ionic bond) ~150 nm, MNPs[CMC]MTX ~240 nm, and MNPs[APTES]MTX (amide bond) ~220 nm. Considering the relatively high polydispersity indexes (a sample’s heterogeneity based on size) ranging from 0.3 to 0.4, these large hydrodynamic diameters could also result from a certain degree of nanoparticle agglomeration.

### 2.6. Investigation of MTX Release from MNPs[APTES]MTX and MNPs[CMC]MTX (Ionic Bond)

**Figure 7:**
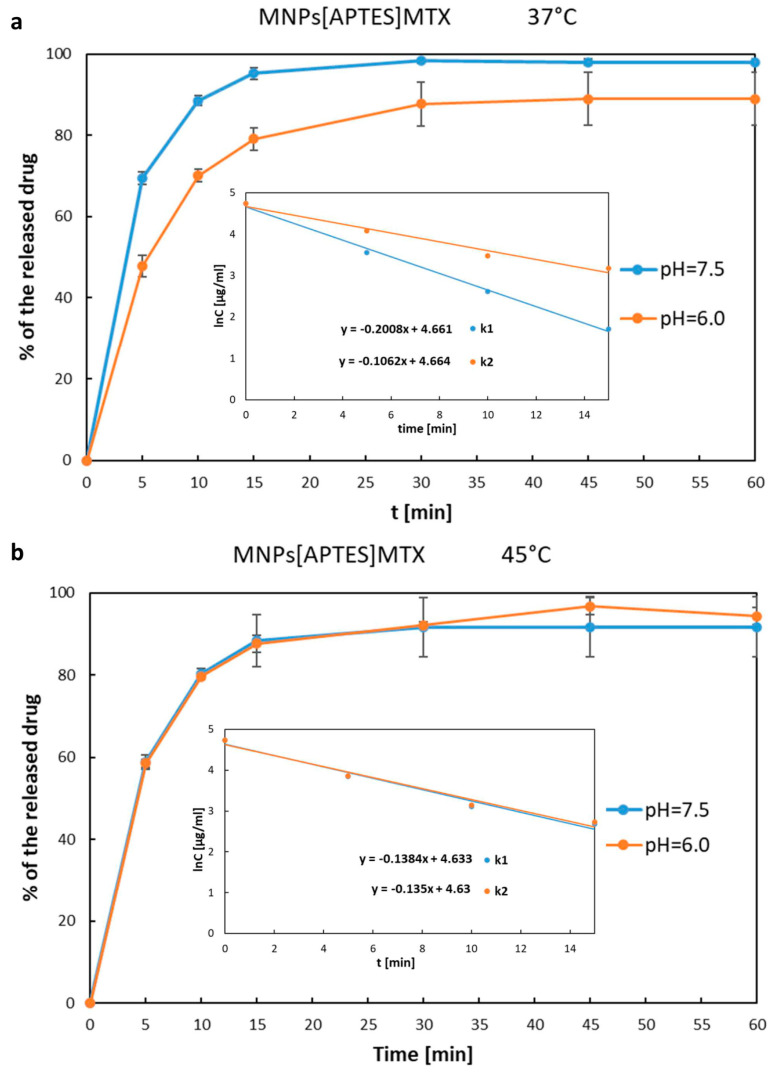
MTX release curves from MNPs[APTES]. Internal graphs present sections corresponding to first-order kinetics representing the equation lnC = −kt + lnC_0_, where C_0_ is the concentration corresponding to the initial amount of MTX on the nanoparticles (t = 0), while C is the drug concentration corresponding to the amount of the drug on the MNPs after the release time t. The slopes indicate the release rate constants: (**a**) k1—release constant for 37 °C, pH = 7.5; k2—release constant for 37 °C, pH = 6.0; (**b**) k1—release constant for 45 °C, pH = 7.5; k2—release constant for 45 °C, pH = 6.0. The experiments were repeated at least three times.

**Figure 8:**
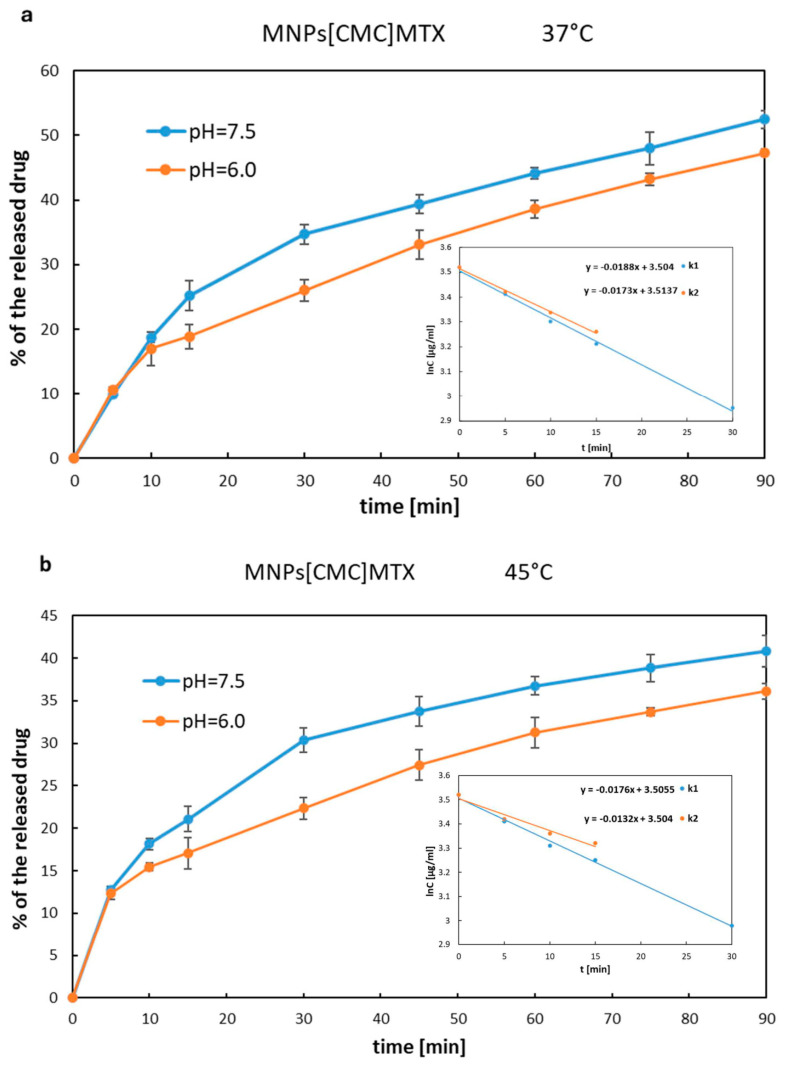
MTX release curves from MNPs[CMC]. The internal graphs present sections corresponding to first-order kinetics representing the equation lnC = −kt + lnC_0_, where C_0_ is the concentration corresponding to the initial amount of MTX on the nanoparticles (t = 0), while C is the drug concentration corresponding to the amount of the drug on the MNPs after the release time t. The slopes indicate the release rate constants: (**a**) k1—release constant for 37 °C, pH = 7.5; k2—release constant for 37 °C, pH = 6.0; (**b**) k1—release constant for 45 °C, pH = 7.5; k2—release constant for 45 °C, pH = 6.0. The experiments were repeated at least three times.

The release rate of MTX immobilized on MNPs[APTES] by ionic bonds is shown in Figure 7a,b as the percentage increase of the drug in solution, where the total amount of MTX immobilized on a given number of nanoparticles was considered as 100%. The drug was rapidly released from MNPs[APTES] according to first-order kinetics for all pH and temperature conditions. After 15 min at 37 °C, approx. 95% (pH = 7.5) or 80% (pH = 6.0) of the total amount of the drug was released, while at 45 °C, approx. 88% of the initial amount of MTX bound on MNPs[APTES] was found in the supernatant for both pH values. In the case of the drug bound with MNPs[CMC], the MTX release process was much slower and the percentage values of the released drugs at 37 °C were as follows: at pH = 7.5 approx. 35% within the first 30 min (first order kinetics,) and approx. 50% after the next 60 min (zero order kinetics starting from the 30th minute of the release); at pH = 6.0 less than 20% within the first 15 min (first-order kinetics) and approx. 47% after the next 60 min (zero order kinetics starting from the 15th minute of the release). The results of the release rate measurements at 45 °C were read from the chart (Figure 8a,b) as follows: at pH = 7.5 approx. 30% release of the total amount of the drug within the first 30 min (first-order kinetics) and approx. 40% after the next 60 min (zero order kinetics starting from the 30th minute of the release); at pH = 6.0 approx. 17% within the first 15 min (first-order kinetics) and approx. 36% after the next 60 min (zero order kinetics starting from the 15th minute of the release). In conclusion, the drug release rates for both nanocarriers (MNPs[APTES] and MNPs[CMC], respectively) were lower in a slightly acidic environment (pH = 6.0) and at higher temperature (45 °C).

The release rates were measured as the wastage of the drug from the nanoparticles using the following equations:

$$lnC=-kt+lnC_{0}\;(\text{first-order kinetics})\;\mathrm{and}\;C=k_{0}\times \Delta t\;(\text{zero order kinetics})$$

where C_0_ is the total amount of the immobilized MTX on the MNPs[APTES] surface at t = 0 min. The release rate constants were determined and summarized in Table 2.

### 2.7. Investigation of MTX Release from MNPs[APTES]MTX (Amide Bond)

The release of MTX bound on nanoparticles via an amide bond was performed at three pH values: 7.5, 6.0, and 2.0, which reflect the environmental conditions of blood serum, tumor intercellular matrix, and intracellular lysosomes, respectively. A common phenomenon in tumours is ion pump disruption [37]. Cancer cells remove H^+^ ions to a greater extent than normal cells, which causes an increase in pH inside cells and a decrease in the extracellular matrix. In turn, in intracellular lysosomes, which during normal cellular metabolism are responsible for the breakdown of proteins and other exogenous materials, the pH is very low. Following receptor-mediated endocytosis, nanoparticles are transported to early endosomes, fusing with lysosomes.

**Figure 9:**
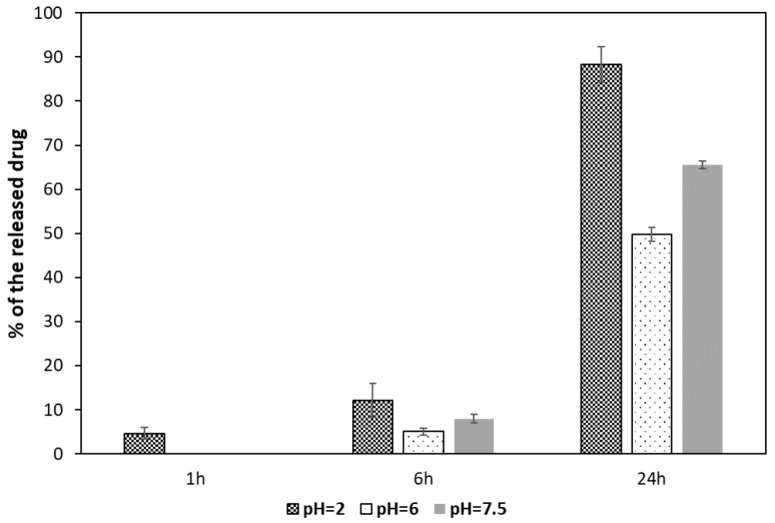
MTX release from MNPs[APTES] (amide bond). The experiments were repeated at least three times.

The results of MTX release bound to MNPs[APTES] by amide bonds are shown in Figure 9.

After the first hour of incubation with the enzyme, the degree of hydrolysis of the amide bond was negligible. At the lowest pH = 2.0, approximately 5% of the drug was released. After 6 h, the supernatant contained 12% of the total amount of bound methotrexate at pH = 2.0, while at pH = 6.0 and 7.5, approximately 5% and 7%, respectively. After a full day of incubation, almost the entire amount of MTX was released (about 89%) in the environment with the lowest pH, whereas in the supernatant at pH = 6.0, about half of the total bound MTX was found, and 66% at pH = 7.5.

### 2.8. The Morphology of Cell Model Grown in a 2D and 3D Condition

**Figure 10:**
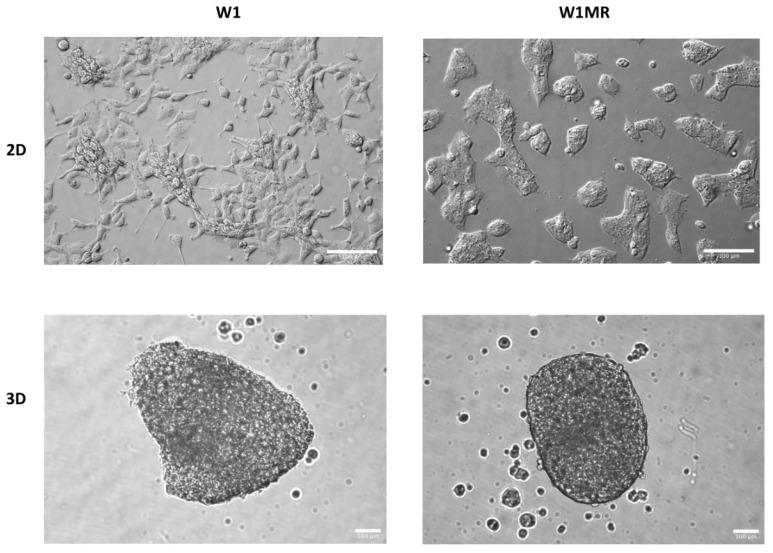
Morphology of drug-sensitive W1 and methotrexate-resistant W1MR cell lines growing in 2D and 3D cell culture conditions. Scale bar = 100 µm.

To check the effect of the obtained forms of cytostatics, in this study, we used the W1 ovarian cancer cell line, derived from the ovarian cancer tissue of an untreated patient, along with the methotrexate-resistant W1MR variant derived from this line. We observed that both kinds of cells grew as a monolayer in the 2D culture method. We could identify epithelial-like cells in W1 and W1MR based on their morphology. These cells were polygonal in shape and grew attached to a substrate in a coherent colony. Likewise, both cell lines formed spheroids using the 3D culture method. W1 and W1MR cell lines formed similar round-type tightly packed compact spheroids with regular edges (Figure 10).

### 2.9. The Analysis of Methotrexate-Sensitive and Methotrexate–Resistant Cell Lines Viability to Methotrexate Conjugated with Nanoparticles in 2D Culture Condition

**Figure 11:**
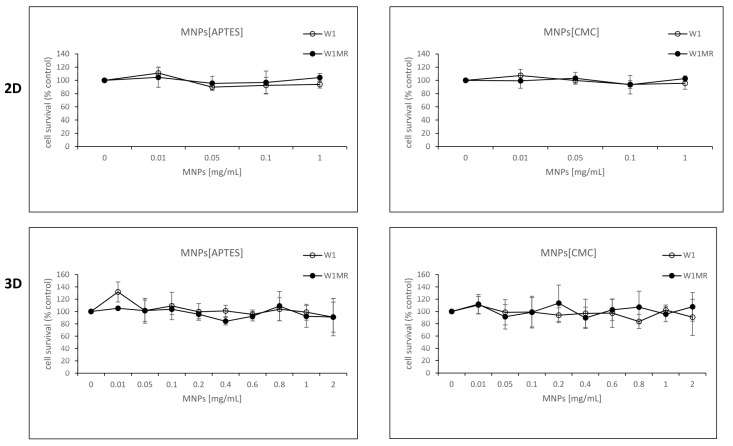
Survival assay of MTT cells in 2D and 3D cell culture conditions. Cell lines W1 and W1MR were seeded at a density of 10,000 cells/well in 96-well plates and treated with increasing concentrations of MNPs[APTES] or MNPs[CMC] at 37 °C for 72 h and cell viability was determined. The experiments were repeated at least three times. The level of viability is shown as a percentage of untreated control cells (mean ± SEM).

In all experiments, MNP[CMC] and MNPs[APTES] (without methotrexate) were tested each time as a control for the action of the nanoparticles themselves (Figure 11). Cell viability in the case of both MNP[CMC] and MNPs[APTES] after 72 h exposure remained at about 100%, predisposing them as good magnetic drug carriers.

**Figure 12:**
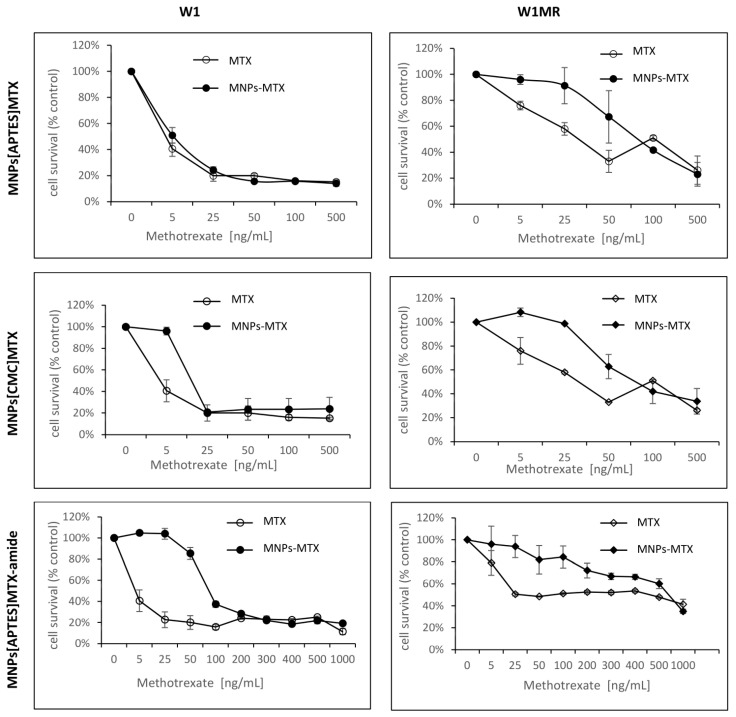
Survival assay of MTT cells in a 2D cell culture condition. Cell lines W1 and W1MR were seeded at a density of 10,000 cells/well in 96-well plates and treated with increasing concentrations of free MTX and conjugated with nanoparticles MNPs[CMC]MTX, MNPs[APTES]MTX or MNPs[APTES]MTX-amide at 37 °C for 72 h and cell viability was determined. The experiments were repeated at least three times. The level of viability is shown as a percentage of untreated control cells (mean ± SEM).

For 72 h, both cell lines grew with increased concentrations of APTES-coated and CMC-coated nanoparticles conjugated with methotrexate by ionic or amide bonds. The obtained results compared to free methotrexate are shown in Figure 12 and Table 3. For all tested nanoparticles conjugated with methotrexate, we observed decreased cell survival in response to increasing methotrexate concentrations but with some differences in the responsive curves.

Treating W1 cells with increasing concentrations of APTES-coated or CMC-coated nanoparticles conjugated with methotrexate by ionic bonds decreased the cell viability similar to free methotrexate The IC_50_ value analysis showed similar resistance to 7.74 ng/mL, with 17.10 ng/mL adequately comparable to 4.29 ng/mL for free methotrexate. Moreover, the response curves in 2D conditions for these forms of the drug are almost identical.

The IC_50_s for W1MR cell lines of free methotrexate and APTES-coated and CMC-coated nanoparticles conjugated with methotrexate by ionic bonds are much higher, 430.69 ng/mL, 448.69 ng/mL, and 409.62 ng/mL, respectively (Table 3). This is a significant increase of resistance around 100, 58, and 24-fold compared to the drugs on the sensitive cell line W1. Although the IC_50_ is similar, the response curves of the W1MR cell line to the nanoparticles conjugated with methotrexate by ionic bonds compared to free methotrexate are different. Nanoparticles at lower concentrations of MTX appear to be less toxic than free methotrexate.

Concerning the APTES-coated nanoparticles conjugated to methotrexate with stronger amide bonds, we observed a significantly higher IC_50_ value for the W1 cell line. It was 86.48 ng/mL and the resistance of the W1 cell line was 21.5-fold lower than for free methotrexate. Analysing the response curve, we observed the characteristic “eye” in both lines, suggesting that nanoparticles at lower concentrations appear less toxic than free methotrexate. For the W1MR cell line, we observed an eight-fold significant increase in resistance for APTES-coated nanoparticles conjugated to methotrexate with amide bonds in comparison to the sensitive W1 cell line (697.05 ng/mL vs. 86.48 ng/mL, respectively). In this case, the IC_50_ for nanoparticles was also significantly higher than for free methotrexate (697.05 ng/mL vs. 430.68 ng/mL). This difference is statistically significant.

In summary, the bonding of methotrexate by ionic bonds causes a similar response in sensitive and resistant cell lines. The stronger amide bonding of the drug to the nanoparticles seems to result in less sensitivity to this form of the drug in both sensitive and resistant lines in this culture model.

### 2.10. The Analysis of Methotrexate-Sensitive and Methotrexate–Resistant Cell Lines Viability to Methotrexate Conjugated with Nanoparticles in 3D Culture Methods

**Figure 13:**
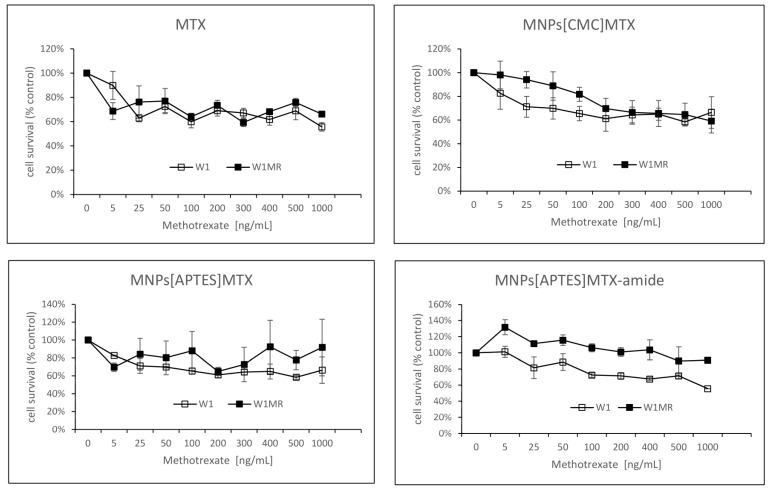
Survival assay of MTT cells in a 3D cell culture condition. Cell line W1 and W1MR were seeded at a density of 25,000 cells/well in 96-well plates and treated with increasing concentrations of MTX, MNPs[CMC]MTX, MNPs[APTES]MTX or MNPs[APTES]MTX-amide at 37 °C for 72 h and cell viability was determined. The experiments were repeated at least three times. The level of viability is shown as a percentage of untreated control cells (mean ± SEM).

In the next step, we examined the response to methotrexate conjugated with nanoparticles of the same cells growing as a spheroid. After the formation of spheroids, the cells were treated with the three different methotrexate nanocarriers for 72 h. The experimental results are presented in Figure 13.

As it turned out, in 3D conditions, both cell lines were resistant to methotrexate at all concentrations tested (up to 1000 ng/mL). The cell viability was reduced max to 60%. The response to methotrexate conjugated by ionic bonds with nanoparticles was very similar and, in these cases, cell viability remained at 60% almost from the beginning of the tested concentrations of MTX for both the W1 and the W1MR cell lines.

A similar drug concentration-dependent response was also observed in the W1 cell line in response to APTES-coated nanoparticles conjugated with methotrexate by stronger amide bonds (Figure 13). The cell viability remained at a level of approximately 60% in the range of methotrexate concentrations tested. The W1MR cell line remained more resistant to this form of drug and was around 90% in the highest tested concentration of MTX. We could not determine the IC_50_ in any of the cell lines either for free methotrexate or for any form of the nanoparticle-bound drug being tested. In 3D culture conditions, it was impossible to overcome the drug resistance of both the sensitive and resistant methotrexate lines with the tested concentrations of methotrexate.

## 3. Discussion

In the above-presented studies, two potential methotrexate nanocarriers based on a magnetic iron oxide Fe_3_O_4_ core were proposed and tested. Magnetic nanoparticles (MNPs) were functionalized with (3-aminopropyl)triethoxysilane (APTES) or N-carboxymethylchitosan (CMC) as biocompatible polymers and donors of amine or carboxyl groups, respectively. These 10-nanometre structures (TEM)—MNPs[APTES] and MNPs[CMC]—turned out to be non-toxic in vivo tests (MTT assay) towards the tested cell lines, which makes them potentially promising drug carriers in, i.e., anticancer therapies. What is worth emphasizing is that one of the main mechanisms of MNPs toxicity is considered to be oxidative stress generated by the release of iron ions involved in the Haber–Weiss, Fenton, and Fenton-like reactions which are believed to be the main processes generating reactive oxygen species (ROS) such as superoxide anions, hydroxyl radicals, and hydrogen peroxides [28]. However, several reports have demonstrated the selectivity of MNPs’ action for tumor and normal cells regarding the level of ROS formation. For example, Ahamed et al. have shown selectively induced apoptosis in cancer cells (HepG2 and A549) via the p53 pathway by MNPs with no toxicity to normal cells [38]. In another study, it was reported that exposure to MNPs caused a rise in the production of ROS and reduced the activity of succinate dehydrogenase in complex II of the mitochondria obtained from cancerous oral tongue squamous cells without significant effects on the control mitochondria [39]. Thus, magnetic iron oxide nanoparticles constitute favourable platforms for drug delivery.

Our VSM magnetism studies showed a strong magnetic susceptibility of the nanocarriers we designed, making them effective tools for targeted therapy by guiding an external magnetic field to the tumor site. This innovative approach not only significantly reduced the toxicity of the chemotherapeutic agent for the entire body but also the MNPs[APTES] and MNPs[CMC] demonstrated a significant hyperthermic effect, generating a temperature increase of up to 45 °C in just a few minutes. This enables the combination of two strategies to destroy the tumour: targeted chemotherapeutic agent delivery and hyperthermia.

MTX attached to APTES- and CMC-coated nanoparticles by ionic bonds demonstrated effective release from the nanocarriers, which is generally necessary for the drug to act once it reaches the tumour. The release rate of MTX from MNPs[CMC] was moderate, which allows this nanocarrier to be considered suitable for controlled drug release. Additionally, deploying MNPs[CMC] would avoid the loss of the drug before it reached the target site and, at the same time, would result in the effective accumulation of the released, unbound drug in the tumor area. On the other hand, the release of MTX from MNPs[APTES] was rapid, which would probably lead to the loss of a significant amount of the chemotherapeutic agent, e.g., in the blood serum, while being delivered to the tumor by guidance with an external magnetic field. However, many favourable features of this carrier, such as ease of synthesis and functionalization, high efficiency of MTX attachment, and lack of toxicity in vitro tests, make it worth considering a modification of the MNPs[APTES]MTX formulation to avoid loss of the drug during delivery. An interesting solution seems to be, for example, the encapsulation of MNPs[APTES]MTX in liposomes. Indeed, using MNPs[APTES] as a carrier appears appropriate if it is desired to obtain high drug concentrations quickly. In the case of MTX conjugated with MNPs[APTES] by a solid amide bond, enzymatic activity is required to release the drug. In our conditions, under the in vitro test (MTT assay), due to the lack of enzymes in the medium, only at higher concentrations of MNPs[APTES]MTX and their accumulation were they were transported inside the tested cells, where the amide bond was enzymatically hydrolysed. In turn, this resulted in the inhibition of the viability of the cell lines.

In summary, binding MTX to nanoparticles via ionic bonding will result in rapid drug release and its accumulation within the tumour. However, there is a risk of losing some of the active compounds in the bloodstream before it reaches the target site. This problem can be avoided by binding MTX via a stable amide bond, but in that case, the release of the drug within the tumour requires proteolytic enzymatic activity.

Drug resistance in cancer, a complex phenomenon that leads to the failure of therapies, is a significant challenge in the field. Our research evaluated the response to anticancer methotrexate conjugated with magnetic nanoparticles in different formulations in drug-sensitive and methotrexate-resistant ovarian cancer cell lines. The 2D cell culture model is well-known to scientists. In this model, cells adhere to the surface and are exposed equally to anticancer drugs. Nevertheless, it is not representative of the complexity of tumor tissue, where a dense cellular structure and interactions between cells and ECM components may influence drug resistance [40]. We observed a typical concentration-dependent response for methotrexate conjugated with nanoparticles by ionic bonds in 2D cell culture conditions. This resulted in decreased cell viability with increased drug concentrations. A different curve was obtained for the tested form of the methotrexate bound to nanoparticles with a stronger amide bond. The drug-sensitive W1 cell line was not sensitive to this form of methotrexate at lower concentrations. Moreover, the methotrexate-resistant cell line was not sensitive to low concentrations of free methotrexate or methotrexate conjugated with magnetic nanoparticles. Different formulations of methotrexate had different levels of drug resistance. The toxicity of methotrexate bound to nanoparticles by an ionic bond, either by APTES or CMC, was similar to that of free methotrexate. Binding the drug to nanoparticles with a stronger amide bond resulted in a weaker response to the drug by methotrexate-resistant cells under the 2D conditions.

In 2D conditions, resistance to cytotoxic drugs is related to cell-specific mechanisms (mainly the expression of membrane transporters). In contrast, in 3D conditions, both cell- and tissue-specific resistance mechanisms (cell density and packing, drug availability) are involved in this process—the change in culture conditions from 2D to 3D causes dramatic changes in drug resistance. Our results indicate that the drug-sensitive and drug-resistant cells cultured in 3D conditions exhibit higher resistance than the cells cultured in 2D conditions against all tested by us formulations of methotrexate: free and conjugated with nanoparticles. According to recent data, 3D models of cell cultures have characteristic features that affect the development of drug resistance. None of the forms of methotrexate we investigated overcame the drug resistance of ovarian cancer cells in the 3D model we tested. Both the W1 and W1MR cell lines formed very similar dense spheroids. There were similarities in their response to free MTX. The W1 and W1MR spheroids were sensitive, beginning from low MTX concentrations, which reduced spheroid viability from 80% in the control to a maximum reduction of 60% in the control at concentrations of 1000 ng/mL of MTX. Nevertheless, further increases in the MTX concentration in both lines did not reduce their viability. This could be due to the insensitivity of quiescent cells in the medium zone of the spheroid to MTX. A further possible reason is the limited properties of MTX to diffuse through a dense cellular structure, which is related to the size of the spheroids. This theory was proposed by West et al. [41] in patients with primary osteosarcoma with different responsiveness to MTX.

The effectiveness of drugs in spheroids depends on various factors such as the size and structure of the spheroid (dense or loose), the cell type (necrotic, quiescent, or proliferating cells), drug concentration, drug diffusion into the dense cellular/ECM structure, and the expression of drug resistance genes and proteins encoded by these genes. Given the organization of tumor tissue, drugs associated with magnetic nanoparticles appear to have the potential to penetrate into the tumor and then be released, resulting in an increased toxicity effect against tumor cells.

Not surprisingly, methotrexate bound to nanoparticles by an ionic bond released rapidly in the environment acts similarly to free methotrexate. Nevertheless, we see more significant potential in drugs associated with magnetic iron oxide nanoparticles with a stronger amide bond. The results indicate that MNPs-conjugated drug in lower doses has a low toxic effect on ovarian cancer cells, and their IC_50_ is 20 times higher than that of free methotrexate in 2D conditions. In 3D conditions, its result is similar to other drug forms on the sensitive line and 20% weaker on the methotrexate-resistant line. However, we are convinced that the weaker result of the MNPs-conjugated MTX was caused by the necessity to penetrate the cells, where the enzymatic activity ensured the amide bond hydrolysis. Nevertheless, in the natural tumor environment, the high proteinase activity would guarantee an effective drug release and thus provide a high cytotoxicity effect. Moreover, drug nanocarriers guided by an external magnetic field and accumulated within the tumor would limit the overall toxicity to the body that an unbound chemotherapeutic would cause. If we additionally consider the phenomenon of hyperthermia, magnetic nanocarriers constitute a much better alternative than the classically used form of the drugs. Understanding how drug resistance mechanisms occur using spheroids may help to develop more effective anticancer therapies. The key is to use the best model in the study. These studies require further research on a highly complex and modified 3D model that considers not only spheroids of a similar size and density as tumours. It is also important to create a research model that considers the components of the extracellular matrix (ECM) that contain the necessary proteinases, the fibroblasts that are secreting transforming growth factors, effective angiogenic stimulators, and changes in pH inside the tumours.

Our findings correspond with some reports on the cytotoxicity of methotrexate conjugated to magnetic nanoparticles. For example, Mohammadi-Samani et al. tested the cytotoxicity of methotrexate immobilized on chitosan-coated MNPs towards SK-BR-3 cell lines [42]. The nanostructures were 10 nm in size, while the average hydrodynamic diameter was 152 nm. The in vitro cell toxicity data showed comparable toxicity in MTX-loaded chitosan-coated MNPs with MTX solution at the same concentrations. Notably, the results of some studies confirmed our assumption that methotrexate conjugated with MNPs in combination with the hyperthermic effect would demonstrate more significant anticancer activity than the free drug. The in vitro cytotoxicity (MTT assay) of MTX loaded on N-isopropylacrylamide (NIPAM) and 2-(N,N-diethylaminoethyl) methacrylate (DEAEMA)—coated MNPs on MCF-7 breast cancer cells was demonstrated in one of the reports [43]. The findings indicated that, in terms of antitumor activity, the utilization of MTX-loaded MNPs with magnetic hyperthermia would be more efficacious than MTX-loaded nanoparticles alone or combined with just water-based hyperthermia.

There are also reports in which the presented results indicate an even more substantial cytotoxic effect of magnetic nanostructures with methotrexate than our findings. For example, Deda et al. studied MNPs modified with glycerol phosphate and phosphorylethanolamine, using the MCF-7 cancer cell line as a model [44]. The results indicated that the conjugation of MTX with magnetite nanoparticles dramatically enhanced its cytotoxicity and decreased the IC_50_ value against MCF-7 cells as compared to the free drug. In another study, the cytotoxicity of MTX immobilized in a hydrogel nanocomposite based on montmorillonite (MTT), Fe_3_O_4_, and alginate (Alg) on MDA-MB-231 breast cancer cells was tested [45]. It was shown that MMT-Fe_3_O_4_-MTX-Alg significantly reduced the percentage of viable cells, with a high down-regulation of the expression level of the anti-apoptotic gene (Bcl-2), and up-regulation of the apoptotic gene (Bax).

We believe that the differences between our results and those of previous reports may result from the fact that cytotoxicity can be influenced by many factors, such as the size of the nanoparticles, the coating molecules, the way the drug is bound and its release kinetics, and particularly the types of cells tested. However, the discovered outcomes support the effectiveness of MNPs as drug delivery platforms and encourage further in-depth investigation.

## 4. Materials and Methods

### 4.1. Synthesis of Magnetic Fe_3_O_4_ Nanoparticles (MNPs)

MNPs were synthesized using the chemical co-precipitative method [46]. However, the method was modified in terms of the reaction temperature as well as in terms of the concentration of the used ammonia solution. In the typical experimental procedure, 8.11 g of iron (III) chloride hexahydrate (FeCl_3_·6H_2_O) and 5.96 of iron (II) chloride tetrahydrate (FeCl_2_·4H_2_O) were diluted in 100 mL of distilled water and placed in a three-necked flask. The mixture was vigorously stirred using a mechanical stirrer, heated to 70 °C, and purged with N_2_. Next, the ammonia solution (NH_3_·H_2_O) was slowly added (1 drop per 1–2 s) to bring to pH = 9.0. The black MNPs precipitate was washed three times with distilled water and then with methanol. Subsequently, the MNPs were suspended in 200 mL of 50 mM TMAOH and sonicated for 1 h in an ultrasonic cleaner. The black MNPs precipitate was pulled away by a magnet, washed with distilled water and methanol, and dried in a drying chamber at 50 °C for 12 h.

### 4.2. Surface Modification of Nanoparticles with (3-Aminopropyl) Trimethoxysilane (APTES) and N-Carboxymethyl Chitosan (CMC)

MNPs functionalization with APTES was carried out using the method of Cao et al. [47]. Briefly, 600 mg of MNPs was dispersed into a mixture of 50 mL distilled water and 75 mL of isopropanol by ultrasonic vibration for 1 h. Then, 30 mL of concentrated (25%) NH_3_ and 1.3 mL of APTES were added to the mixture under constant mechanical stirring and heating (55 °C) for 20 h. After the reaction, the brown precipitate was washed three times with water and then with methanol and dried at 35 °C for 24 h in a drying chamber.

MNPs functionalization with CMC was carried out using the method designed. First, 600 mg MNPs was suspended in the mixture of 75 mL isopropanol and 50 mL distilled water and sonicated in an ultrasonic cleaner for 1 h. Then, 30 mL of distilled water was added to 300 mg CMC and the mixture was stirred until complete dissolution. The CMC solution was added to the suspension of MNPs and sonicated for the next 30 min. Next, the mixture was stirred using a mechanical stirrer at 55 °C for 20 h. After the reaction, the brown precipitate was washed three times with water and then with methanol and dried at 35 °C for 24 h in a drying chamber.

### 4.3. Characterization with FT-IR and Transmission Electron Microscope (TEM) Imaging of APTES- and CMC-Modified Nanoparticles (MNPs[APTES] and CMC[APTES])

The nanoparticles MNPs[APTES] or MNPs[CMC], 2 mg, were added to 200 mg of KBr and ground in a mortar. Then, a small sample of the mixture was pressed using a hydraulic press Sirio (Mikran, Poznań, Poland) into a pellet. Fourier transform infrared (FT-IR) spectra were acquired using an IR Spirit (Shimadzu, Shim-Pol, Warszawa, Poland) spectrometer in the range of 4000–400 cm^−1^. TEM images were acquired using a transmission microscope Titan G2 60-300 (FEI, Eindhoven, The Netherlands).

### 4.4. Magnetic VSM Properties Measurements of MNPs[APTES] and MNPs[CMC]

Magnetometry measurements were made using a vibration magnetometer type 7407 from LakeShore Cryotronics (Westerville, OH, USA) equipped with a liquid nitrogen-cooled flow cryostat. Measurements of the temperature dependence of the magnetization curves were made for each sample during magnetization at the selected temperatures. The first type of measurement (MvT) allows the examination of the magnetic nature of the samples; in particular, the estimation of the blocking temperature (T_b_) based on the inflection point and maximum of the M_ZFC_(T) curve (ZFC—Zero Field-Cooled) [48]. The blocking temperature is defined as the transition temperature from the blocked state magnetic moments to the superparamagnetic state. At the blocking temperature, the hysteresis loop disappears because the thermal fluctuations of the magnetic moments are large enough that the average magnetization without an external magnetic field is zero. The transition from the locked state to the superparamagnetic state usually occurs at one critical point temperature characteristic of a given system. MvT measurements were performed using the following sequence: (i) heating the sample to T = 440 K in H = 0, (ii) cooling the sample to T = 90 K in H = 0, (iii) measurement of the ZFC relationship while heating the sample in a constant field H = 100 Oe, (iv) measurement of the relationship FC (FC—Field-Cooled) when cooling the sample in a constant field H = 100 Oe.

The second type of measurement (MvHvT) allows for the estimation of temperature dependencies of saturation magnetization (based on the approach to saturation model [49]) and the remanence and coercive field, i.e., parameters indicating the expected superparamagnetic nature of the tested materials. Full hysteresis loops were tested in a magnetic field strength range up to 16.5 kOe. The loops were collected at temperatures T = 100, 120, 140, 170, 200, 250, 300, 360, and 440 K.

### 4.5. Hyperthermic Properties of MNPs[APTES] and MNPs[CMC]

Hyperthermal properties of nanoparticle solutions (4 mg/mL) were studied using induction equipment heating by DACPOL (Piaseczno, Poland). The control system was equipped with a transistor generator (by AMBRELL, Hengelo, The Netherlands) of high frequency to induce induction heating (EASY Heat 0224FFCE) with a power of up to 2.4 kW, a component power supply system, and a system controlling the current, power, and sample heating time. The cooling system (TEXA TCW12NBSBCP0000) was connected to the generator and the induction coil. The generator converts the supply voltage of 240 V with a frequency of 50 Hz into the voltage of frequencies in the 150–450 kHz range. The record of temperature increase as a function of time (under the influence of an external electromagnetic field, at given current strengths) was carried out from a temperature of 36.6 °C to 45 °C.

### 4.6. Attaching Methotrexate (MTX) to the APTES-Coated and CMC-Coated MNPs by Ionic Bonds

First, 600 mg of MNPs[APTES] or MNPs[CMC] was suspended in 60 mL of phosphate buffer (pH = 6.0) and sonicated in an ultrasonic cleaner for 30 min with simultaneous cooling. Then, 30 mg of MTX was dissolved in the mixture of phosphate buffer (pH = 6.0) and DMSO (1:1, 30 mL). Next, the solution of MTX was slowly instilled into the suspension of nanoparticles during continuous sonication. The suspension containing MTX was sonicated for the next 15 min. After that, the mixture was stirred using a mechanical stirrer for 20 h at room temperature. The nanoparticles were pulled away by a magnet, washed with distilled water (2 × 60 mL) and methanol (2 × 60 mL), and dried at 30 °C for 24 h in a drying chamber.

The method used for the MTX loading capacity quantitation made use of the fact that KHSO_4_ causes the complete removal of the drug from its salts with APTES or CMC. The basics of the method were previously reported [50]. Briefly, a sample of MNP[APTES]MTX or MNPs[CMC]MTX (5 mg) was suspended in 2 mL of the mixture of 0.1 M KHSO_4_ (pH = 1.5) and DMSO (4:1) and sonicated for 15 min in an ultrasonic cleaner. After separating the nanoparticles (MNP[APTES] or MNPs[CMC]) with a magnet, the solution was centrifugated (13,000 rpm, 10 min, 25 °C) and the concentration of the released drug in the supernatant was measured using a UV–vis spectrophotometer (2450 UV-VIS Shimadzu, SHIM-MED, Warszawa, Poland) at wavelength λ = 370 nm. The measurements were repeated in three independent trials with satisfactory reproducibility of the results.

### 4.7. Attaching Methotrexate (MTX) to the APTES-Coated MNPs by an Amide Bond

To conjugate the MTX on the surface of the MNPs[APTES] by an amide bond, 1 g of MNPs[APTES] was dispersed in a mixture of 50 ml of DMSO and 10 mL of distilled water and sonicated for 20 min in an ultrasonic cleaner. Then, 50 mg of free MTX, 105 mg of EDC hydrochloride, and 63.2 mg of N-hydroxysuccinimide (NHS) were dissolved in 20 mL of DMSO, and the mixture was added to the suspension of nanoparticles. The pH of the solution was adjusted to 8.2 by the triethylamine addition. The resulting suspension was stirred overnight (20 h) at room temperature (~22 °C) in the dark. Following MTX conjugation, the modified nanoparticles were isolated with an external magnet, washed with distilled water (2 × 60 mL), and methanol (2 × 60 mL), and dried at 30 °C for 24 h in a drying chamber.

The MTX loading capacity quantification was performed using proteinase K. Proteinase K was dissolved in 2 mL of PBS buffer (pH = 7.5) to obtain a final enzyme concentration equal to 1 mg/mL. The mixture was added to the nanoparticles (5 mg) and incubated at 37 °C for 3 h. After that time, the solution was centrifuged (13,000 rpm, 10 min, 25 °C), and the concentration of the released drug in the supernatant was measured using a UV–vis spectrophotometer (2450 UV-VIS Shimadzu, SHIM-MED, Warszawa, Poland) at wavelength λ = 370 nm. The measurements were repeated in three independent trials with satisfactory reproducibility of the results.

### 4.8. DLS and Zeta Potential Measurements of MNPs, MNPs[APTES], MNPs[CMC] and MTX-Functionalized Nanoparticles (MNPs[APTES]MTX and MNPs[CMC]MTX)

The hydrodynamic size distribution was measured by the dynamic light scattering (DLS) method using a Zetasizer Nano-ZS instrument Malvern (A.P.I. Instruments, Warszawa, Poland). Samples were suspended in buffer (pH = 7.5, 0.1 mg/mL) and measured 3 times with 15 runs at 25 °C. The zeta potential was measured by laser Doppler velocimetry using the same instrument. Samples were suspended in buffer (pH = 7.5, 0.1 mg/mL) and measured 3 times with 30 runs. The composition of the buffer corresponded to the composition of RPMI 1640 medium (Ca(NO_3_)_2_ × 4H_2_O 0.1 g/L, KCl 0.4 g/L, MgSO_4_ × 7H_2_O 0.1 g/L, NaCl 6 g/L, NaHCO_3_ 2 g/L, Na_2_HPO_4_ 0.8 g/L, D-glucose 2 g/L); www.capricorn-scientific.com (accessed on 7 January 2024).

### 4.9. Investigation of MTX Release from MNPs[APTES]MTX and MNPs[CMC]MTX (Ionic Bond)

First, 5 mg of MNPs[APTES]MTX (ionic bond) or 20 mg of MNPs[CMC]MTX was dispersed in 2 mL of buffer (pH = 7.5 or 6.0) and incubated at 37 °C or 45 °C for 24 h. At the specified time intervals, nanoparticles were pulled away with a magnet, and the solution after separation was centrifugated (RCF = 17,500× *g*, 5 min, 25 °C). The concentration of released drug in the supernatant was determined on a UV–vis spectrophotometer (2450 UV-VIS Shimadzu, SHIM-MED, Warszawa, Poland) at wavelength λ = 370 nm. To the remaining nanoparticles after sample separation, 2 mL of fresh buffer was added, and the incubation at 37 °C of the sample was continued until the next measurement. Continuous removal of the released drug was necessary; otherwise, the equilibrium state of dissociation of the salt (APTES^+^MTX^−^ or CMC^−^MTX^+^) was reached. The experiments were performed three times with satisfactory reproducibility of the results. The composition of the buffer corresponded to the composition of RPMI 1640 medium (Ca(NO_3_) × 4H_2_O 0.1 g/L, KCl 0.4 g/L, MgSO_4_ × 7H_2_O 0.1 g/L, NaCl 6 g/L, NaHCO_3_ 2 g/L, Na_2_HPO_4_ 0.8 g/L, D-glucose 2 g/L; www.capricorn-scientific.com (accessed on 7 January 2024). The specific pH was adjusted using a pH meter by the addition of sodium acid phosphate (NaH_2_PO_4_ × 2H_2_O, 0.8 g/L).

### 4.10. Investigation of MTX Release from MNPs[APTES]MTX (Amide Bond)

MTX-grafted nanoparticles (10 mg) were suspended in a solution of 0.1 mg/mL protease K in 2 mL of phosphate-buffered saline (PBS, pH = 7.5) and incubated at 37 °C under constant stirring. The solution pH of 2.0 or 6.0 was adjusted by the titration of 1.0 M HCl. Following incubation for 1, 6, and 24 h, the nanoparticle suspensions were centrifuged (RCF = 17,500× *g*, 5 min, 25 °C) and the concentration of released drug in the supernatant was determined on a UV–vis spectrophotometer (2450 UV-VIS Shimadzu, SHIM-MED, Warszawa, Poland) at wavelength λ = 370 nm.

### 4.11. Cell Lines and Cell Culture

In this study, we used the W1 ovarian cancer cell line, established from ovarian cancer tissue from an untreated patient, and the methotrexate-resistant cell line W1MR as described previously [51]. The IC_50_ between the W1 and W1MR cell lines showed a high increase in resistance to MTX. The final concentration of MTX used to select the resistant cells was 28 ng/mL. Both cell lines were harvested in RPMI 1640 medium supplemented with 2 pML-glutamine, FBS (10% *v*/*v*), penicillin (100 units/mL), streptomycin (100 units/mL), and amphotericin B (25 g/mL). Cells were grown at 37 °C in a 5% CO_2_ atmosphere in T25 and T75 flasks (Googlab Scientific, Starogard Gdański, Poland). Cytotoxic drugs, RPMI 1640 medium, and FBS were acquired from Sigma (St. Louis, MO, USA). The antibiotic-antimycotic solution was obtained from Corning (New York, NY, USA).

### 4.12. The Cell Morphology in 2D (Two-Dimensional) and 3D (Three-Dimensional) Cell Culture

For the growth of cells in adherent 2D cell cultures, cells were trypsinized using 0.25% trypsin (Corning), resuspended in 1 mL of the growth medium, and transferred to 6-well plates in an amount of 250,000 cells per well. The cell culture was conducted for five days, and the medium was changed every two days. An inverted microscope was used to evaluate the morphology of the drug-sensitive and resistant cell lines.

In 3D cell cultures, the cells formed spheroids. The cells were trypsinized, resuspended in 200 μL of the growth medium, and transferred to nonadherent surface 96-well plates (BRAND plates inter Grade, F-bottom, 781902 (Merck, Warszawa, Poland), in an amount of 10,000 cell per well. The cell culture was conducted for five days, and the medium was changed every two days. Spheroid morphology of the drug-sensitive and resistant cell lines was evaluated using an inverted microscope (Primovert Zeiss, Pro Foto, Szczecin, Poland).

### 4.13. MTT Assay

The drug sensitivity/resistance was measured using the MTT assay. Initially, cells were seeded in 96-well plates: 10,000 cells per well (2D) and 25,000 for 3D cell culture per well. After 48 h of culture, the medium was replaced with fresh medium with or without free methotrexate (as a positive control), MNPs[CMC] and MNPs[APTES] (as a negative control), and APTES-coated or CMC-coated nanoparticles conjugated with methotrexate by ionic or amid bonds in increasing concentrations, and the cells were incubated at 37 °C in a 5% CO_2_ atmosphere for 72 h. Afterwards, 10 μL of MTT (4 mg/mL) was added, and the cells were incubated for 4 h under the same conditions. After this time, 100 μL of 10% SDS in 0.01 M HCl was added. The cell culture was continued until the following day. The absorbance was measured at 570 nm using a microplate reader with a reference wavelength of 720 nm (Synergy 2 Multi-Detectin Microplate Reader by BioTek Instruments, Inc., Santa Clara, CA, USA) and analysed by Gen5 software (version 2.07.17). We used a sample containing cell culture medium, MTT, and SDS solution without cells as a negative control. The experiments were repeated three times, and each sample with an appropriate nanoparticle concentration was duplicated. For all forms of magnetic nanoparticles conjugated with methotrexate in the investigated cell lines, the IC_50_ value was determined.

### 4.14. Statistical Analysis

The statistical analysis was performed using Excel 2016 software (Microsoft, Redmond, WA, USA). The statistical significance of the differences was determined by applying Student’s *t*-test at *p* < 0.05, *p* < 0.01, and *p* < 0.001.

## 5. Conclusions

We evaluated the potential of the magnetic Fe_3_O_4_ nanoparticles for the targeted delivery of methotrexate to ovarian cancer cells. We designed and synthesized iron oxide (Fe_3_O_4_) nanoparticles functionalized with (3-aminopropyl)triethoxysilane (APTES) or N-carboxymethylchitosan (CMC). The coating polymers provided amino and carboxyl groups to which we attached methotrexate using strong covalent amide bonds, or for the first time reported, ionic bonds.

All obtained nanostructures exhibited significant magnetic and hyperthermic properties. MNPs[APTES] and MNPs[CMC] turned out to be non-toxic (in vitro tests), which makes them potential drug nanocarriers. Methotrexate attached to MNPs[CMC] was released moderately at pH = 6.0 and 7.5 and 37 °C and 45 °C, indicating controlled drug release. The tested methotrexate conjugated with MNPs[APTES] via an ionic bond was released rapidly, which may be an advantage within the tumor, provided that the nanocarrier is delivered in a form that prevents the loss of the drug in the bloodstream. MTX attached to MNPs[APTES] via an amide bond was released effectively at low pH, i.e., in conditions corresponding to the environment inside cell lysosomes.

MTX-immobilized nanoparticles were incubated with ovarian cancer cell lines in monolayer 2D and spheroid 3D conditions of cell culture. The cytotoxicity test results showed similar ovarian cancer cell proliferation inhibition values of methotrexate-bound nanoparticles compared to the free MTX. The conjugation of MTX with nanoparticles gives it an additional magnetic feature.

## Figures and Tables

**Table 2:** Methotrexate release rate constants.

MNPs[APTES]MTX				
	**37 °C**		**45 °C**	
	pH = 7.5	pH = 6.0	pH = 7.5	pH = 6.0
0–60 min I—order kinetics k [min^−1^]	0.2008	0.1602	0.1384	0.1350
**MNPs[CMC]MTX**				
	**37 °C**		**45 °C**	
	pH = 7.5	pH = 6.0	pH = 7.5	pH = 6.0
0–30 min I—order kinetics k [min^−1^]	0.0188		0.0176	
0–15 min I—order kinetics k [min^−1^]		0.0173		0.0132
30–90 min 0—order kinetics k_0_ [µg/mL × min^−1^]	0.197		0.152	
15–90 min 0—order kinetics k_0_ [µg/mL × min^−1^]		0.176		0.134

**Table 3:** A summary of cell line resistance to cytotoxic drug treatment in 2D cell culture conditions. IC_50_ mean is indicated for each drug. ↑ indicate multiplicities of resistance with respect to W1 cell line. *** *p* < 0.001.

	W1	W1MR
MTX	4.29	430.69
	SD ± 0.68	SD ± 25.47
	1	100.4 ↑***
MNPs[APTES]MTX	7.74	448.69
	SD ± 2.60	SD ± 16.69
	1	58.0 ↑***
MNPs[CMC]MTX	17.10	409.62
	SD ± 0.22	SD ± 7.36
	1	24.0 ↑***
MNPs[APTES]MTXAMID	86.48	697.05
	SD ± 2.33	SD ± 55.25
	1	8.1 ↑***

**Table 1:** Summary of SAR and ILP parameters results.

Aqueous Solution of MNPs[APTES] Nanoparticles, C = 4 mg MNPs/mL			
I = 175 A		I = 225 A	I = 250 A
H [kA/m]	27.64	33.83	36.41
f [kHz]	356	357	356
**SAR [W/g]**	**59**	**114**	**154**
**ILP [nH^2^/kg]**	**0.22**	**0.28**	**0.33**
**Aqueous Solution of MNPs[CMC] Nanoparticles, C = 4 mg MNPs/mL**			
I = 175 A		I = 225 A	I = 250 A
H [kA/m]	27.64	33.83	36.41
f [kHz]	356	357	356
**SAR [W/g]**	**208**	**314**	**364**
**ILP [nH^2^/kg]**	**0.76**	**0.77**	**0.77**

## Data Availability

Data are contained within the article.

## References

[1] Bao Y., Sherwood J.A., Sun Z. (2018). Magnetic Iron Oxide Nanoparticles as *T*_1_ Contrast Agents for Magnetic Resonance Imaging. J. Mater. Chem. C.

[2] Huang J., Zhong X., Wang L., Yang L., Mao H. (2012). Improving the Magnetic Resonance Imaging Contrast and Detection Methods with Engineered Magnetic Nanoparticles. Theranostics.

[3] Zhou Q., Wei Y. (2017). For Better or Worse, Iron Overload by Superparamagnetic Iron Oxide Nanoparticles as a MRI Contrast Agent for Chronic Liver Diseases. Chem. Res. Toxicol..

[4] Russell E., Dunne V., Russell B., Mohamud H., Ghita M., McMahon S.J., Butterworth K.T., Schettino G., McGarry C.K., Prise K.M. (2021). Impact of Superparamagnetic Iron Oxide Nanoparticles on In Vitro and In Vivo Radiosensitisation of Cancer Cells. Radiat. Oncol..

[5] Palzer J., Eckstein L., Slabu I., Reisen O., Neumann U.P., Roeth A.A. (2021). Iron Oxide Nanoparticle-Based Hyperthermia as a Treatment Option in Various Gastrointestinal Malignancies. Nanomaterials.

[6] Obaidat I.M., Narayanaswamy V., Alaabed S., Sambasivam S., Muralee Gopi C.V.V. (2019). Principles of Magnetic Hyperthermia: A Focus on Using Multifunctional Hybrid Magnetic Nanoparticles. Magnetochemistry.

[7] Mu X., Li J., Yan S., Zhang H., Zhang W., Zhang F., Jiang J. (2018). siRNA Delivery with Stem Cell Membrane-Coated Magnetic Nanoparticles for Imaging-Guided Photothermal Therapy and Gene Therapy. ACS Biomater. Sci. Eng..

[8] Uskoković V., Tang S., Wu V.M. (2019). Targeted Magnetic Separation of Biomolecules and Cells Using Earthicle-Based Ferrofluids. Nanoscale.

[9] El-Boubbou K. (2018). Magnetic Iron Oxide Nanoparticles as Drug Carriers: Clinical Relevance. Nanomedicine.

[10] Bukowski K., Kciuk M., Kontek R. (2020). Mechanisms of Multidrug Resistance in Cancer Chemotherapy. Int. J. Mol. Sci..

[11] Duan C., Yu M., Xu J., Li B.-Y., Zhao Y., Kankala R.K. (2023). Overcoming Cancer Multi-Drug Resistance (MDR): Reasons, Mechanisms, Nanotherapeutic Solutions, and Challenges. Biomed. Pharmacother..

[12] Fan J., To K.K.W., Chen Z.-S., Fu L. (2023). ABC Transporters Affects Tumor Immune Microenvironment to Regulate Cancer Immunotherapy and Multidrug Resistance. Drug Resist. Updates.

[13] Fletcher J.I., Haber M., Henderson M.J., Norris M.D. (2010). ABC Transporters in Cancer: More than Just Drug Efflux Pumps. Nat. Rev. Cancer.

[14] Correia A.L., Bissell M.J. (2012). The Tumor Microenvironment Is a Dominant Force in Multidrug Resistance. Drug Resist. Updates.

[15] Rahmanian M., Seyfoori A., Ghasemi M., Shamsi M., Kolahchi A.R., Modarres H.P., Sanati-Nezhad A., Majidzadeh-A K. (2021). In-Vitro Tumor Microenvironment Models Containing Physical and Biological Barriers for Modelling Multidrug Resistance Mechanisms and Multidrug Delivery Strategies. J. Control. Release.

[16] Tan Q., Saggar J.K., Yu M., Wang M., Tannock I.F. (2015). Mechanisms of Drug Resistance Related to the Microenvironment of Solid Tumors and Possible Strategies to Inhibit Them. Cancer J..

[17] Padera T.P., Meijer E.F.J., Munn L.L. (2016). The Lymphatic System in Disease Processes and Cancer Progression. Annu. Rev. Biomed. Eng..

[18] Westhoff M.A., Zhou S., Bachem M.G., Debatin K.M., Fulda S. (2008). Identification of a Novel Switch in the Dominant Forms of Cell Adhesion-Mediated Drug Resistance in Glioblastoma Cells. Oncogene.

[19] Garcia-Mayea Y., Mir C., Masson F., Paciucci R., LLeonart M.E. (2020). Insights into New Mechanisms and Models of Cancer Stem Cell Multidrug Resistance. Semin. Cancer Biol..

[20] Nowacka M., Sterzynska K., Andrzejewska M., Nowicki M., Januchowski R. (2021). Drug Resistance Evaluation in Novel 3D In Vitro Model. Biomed. Pharmacother..

[21] Weaver V.M., Lelièvre S., Lakins J.N., Chrenek M.A., Jones J.C.R., Giancotti F., Werb Z., Bissell M.J. (2002). Β4 Integrin-Dependent Formation of Polarized Three-Dimensional Architecture Confers Resistance to Apoptosis in Normal and Malignant Mammary Epithelium. Cancer Cell.

[22] Kapałczyńska M., Kolenda T., Przybyła W., Zajączkowska M., Teresiak A., Filas V., Ibbs M., Bliźniak R., Łuczewski Ł., Lamperska K. (2016). 2D and 3D Cell Cultures—A Comparison of Different Types of Cancer Cell Cultures. Aoms.

[23] Li C., Kato M., Shiue L., Shively J.E., Ares M., Lin R.-J. (2006). Cell Type and Culture Condition–Dependent Alternative Splicing in Human Breast Cancer Cells Revealed by Splicing-Sensitive Microarrays. Cancer Res..

[24] Świerczewska M., Sterzyńska K., Ruciński M., Andrzejewska M., Nowicki M., Januchowski R. (2023). The Response and Resistance to Drugs in Ovarian Cancer Cell Lines in 2D Monolayers and 3D Spheroids. Biomed. Pharmacother..

[25] Lee J., Cuddihy M.J., Kotov N.A. (2008). Three-Dimensional Cell Culture Matrices: State of the Art. Tissue Eng. Part B Rev..

[26] Vinci M., Gowan S., Boxall F., Patterson L., Zimmermann M., Court W., Lomas C., Mendiola M., Hardisson D., Eccles S.A. (2012). Advances in Establishment and Analysis of Three-Dimensional Tumor Spheroid-Based Functional Assays for Target Validation and Drug Evaluation. BMC Biol..

[27] Marushima H. (2011). Three-Dimensional Culture Promotes Reconstitution of the Tumor-Specific Hypoxic Microenvironment under TGFβ Stimulation. Int. J. Oncol..

[28] Wu H., Yin J.-J., Wamer W.G., Zeng M., Lo Y.M. (2014). Reactive Oxygen Species-Related Activities of Nano-Iron Metal and Nano-Iron Oxides. J. Food Drug Anal..

[29] Sabouri Z., Sabouri M., Moghaddas S.S.T.H., Darroudi M. (2022). Design and Preparation of Amino-Functionalized Core-Shell Magnetic Nanoparticles for Photocatalytic Application and Investigation of Cytotoxicity Effects. J. Environ. Health Sci. Eng..

[30] Ghutepatil P.R., Salunkhe A.B., Khot V.M., Pawar S.H. (2019). APTES (3-Aminopropyltriethoxy Silane) Functionalized MnFe2O4 Nanoparticles: A Potential Material for Magnetic Fluid Hyperthermia. Chem. Pap..

[31] Comanescu C. (2023). Recent Advances in Surface Functionalization of Magnetic Nanoparticles. Coatings.

[32] Das S.S., Kar S., Singh S.K., Hussain A., Verma P.R.P., Beg S. (2022). Carboxymethyl Chitosan in Advanced Drug-Delivery Applications. Chitosan in Drug Delivery.

[33] Wildeboer R.R., Southern P., Pankhurst Q.A. (2014). On the Reliable Measurement of Specific Absorption Rates and Intrinsic Loss Parameters in Magnetic Hyperthermia Materials. J. Phys. D Appl. Phys..

[34] Lachowicz D., Górka W., Kmita A., Bernasik A., Żukrowski J., Szczerba W., Sikora M., Kapusta C., Zapotoczny S. (2019). Enhanced Hyperthermic Properties of Biocompatible Zinc Ferrite Nanoparticles with a Charged Polysaccharide Coating. J. Mater. Chem. B.

[35] Kmita A., Lachowicz D., Żukrowski J., Gajewska M., Szczerba W., Kuciakowski J., Zapotoczny S., Sikora M. (2019). One-Step Synthesis of Long Term Stable Superparamagnetic Colloid of Zinc Ferrite Nanorods in Water. Materials.

[36] Maguire C.M., Rösslein M., Wick P., Prina-Mello A. (2018). Characterisation of Particles in Solution—A Perspective on Light Scattering and Comparative Technologies. Sci. Technol. Adv. Mater..

[37] De Milito A., Fais S. (2005). Tumor Acidity, Chemoresistance and Proton Pump Inhibitors. Future Oncol..

[38] Ahamed M., Alhadlaq H.A., Khan M.A.M., Akhtar M.J. (2013). Selective Killing of Cancer Cells by Iron Oxide Nanoparticles Mediated through Reactive Oxygen Species via P53 Pathway. J. Nanopart Res..

[39] Jahanbani J., Ghotbi M., Shahsavari F., Seydi E., Rahimi S., Pourahmad J. (2020). Selective Anticancer Activity of Superparamagnetic Iron Oxide Nanoparticles (SPIONs) against Oral Tongue Cancer Using in Vitro Methods: The Key Role of Oxidative Stress on Cancerous Mitochondria. J. Biochem. Mol. Toxicol..

[40] Xu X., Farach-Carson M.C., Jia X. (2014). Three-Dimensional In Vitro Tumor Models for Cancer Research and Drug Evaluation. Biotechnol. Adv..

[41] West G.W., Weichselbaum R., Little J.B. (1980). Limited Penetration of Methotrexate into Human Osteosarcoma Spheroids as a Proposed Model for Solid Tumor Resistance to Adjuvant Chemotherapy. Cancer Res..

[42] Mohammadi-Samani S., Miri R., Salmanpour M., Khalighian N., Sotoudeh S., Erfani N. (2013). Preparation and Assessment of Chitosan-Coated Superparamagnetic Fe_3_O_4_ Nanoparticles for Controlled Delivery of Methotrexate. Res. Pharm. Sci..

[43] Najafipour A., Gharieh A., Fassihi A., Sadeghi-Aliabadi H., Mahdavian A.R. (2021). MTX-Loaded Dual Thermoresponsive and pH-Responsive Magnetic Hydrogel Nanocomposite Particles for Combined Controlled Drug Delivery and Hyperthermia Therapy of Cancer. Mol. Pharm..

[44] Deda D.K., Cardoso R.M., Kawassaki R.K., De Oliveira A.R., Toma S.H., Baptista M.S., Araki K. (2021). Cytotoxicity of Methotrexate Conjugated to Glycerol Phosphate Modified Superparamagnetic Iron Oxide Nanoparticles. J. Nanosci. Nanotechnol..

[45] Farshi Azhar F., Ahmadinia A., Mousazadeh H., Kheirkhah E. (2024). A pH-Sensitive Magnetic Hydrogel Nanocomposite Based on Alginate for Controlled Release of Methotrexate. Int. J. Polym. Mater. Polym. Biomater..

[46] Massart R. (1981). Preparation of Aqueous Magnetic Liquids in Alkaline and Acidic Media. IEEE Trans. Magn..

[47] Cao H., He J., Deng L., Gao X. (2009). Fabrication of Cyclodextrin-Functionalized Superparamagnetic Fe_3_O_4_/Amino-Silane Core–Shell Nanoparticles via Layer-by-Layer Method. Appl. Surf. Sci..

[48] Bruvera I.J., Mendoza Zélis P., Pilar Calatayud M., Goya G.F., Sánchez F.H. (2015). Determination of the Blocking Temperature of Magnetic Nanoparticles: The Good, the Bad, and the Ugly. J. Appl. Phys..

[49] Zhang H., Zeng D., Liu Z. (2010). The Law of Approach to Saturation in Ferromagnets Originating from the Magnetocrystalline Anisotropy. J. Magn. Magn. Mater..

[50] Nowak-Jary J., Gronczewska E., Worobiec W. (2018). Hampered Binding to Blood Serum Proteins and the Biological Activity of Antimicrobial Peptide Containing N3-(4-Methoxyfumaroyl)-L-2,3-Diaminopropanoic Acid Immobilized on Magnetic Nanoparticles. Pharm. Chem. J..

[51] Sterzyńska K., Kaźmierczak D., Klejewski A., Świerczewska M., Wojtowicz K., Nowacka M., Brązert J., Nowicki M., Januchowski R. (2019). Expression of Osteoblast-Specific Factor 2 (OSF-2, Periostin) Is Associated with Drug Resistance in Ovarian Cancer Cell Lines. Int. J. Mol. Sci..

